# Functional exploration of the glycoside hydrolase family GH113

**DOI:** 10.1371/journal.pone.0267509

**Published:** 2022-04-22

**Authors:** Marie Couturier, Mélanie Touvrey-Loiodice, Nicolas Terrapon, Elodie Drula, Laurine Buon, Christine Chirat, Bernard Henrissat, William Helbert

**Affiliations:** 1 CERMAV, Univ. Grenoble Alpes, CNRS, Grenoble, France; 2 AFMB, UMR 7257 CNRS Aix-Marseille Univ., USC 1408 INRAE, Marseille, France; 3 Biodiversité et Biotechnologie Fongiques, UMR 1163, INRAE, Marseille, France; 4 Institute of Engineering (Grenoble INP), LGP2, Université Grenoble Alpes, CNRS, Grenoble, France; 5 Department of Biological Sciences, King Abdulaziz University, Jeddah, Saudi Arabia; 6 DTU Bioengineering, Technical University of Denmark, Kgs. Lyngby, Denmark; Weizmann Institute of Science, ISRAEL

## Abstract

β-Mannans are a heterogeneous group of polysaccharides with a common main chain of β-1,4-linked mannopyranoside residues. The cleavage of β-mannan chains is catalyzed by glycoside hydrolases called β-mannanases. In the CAZy database, β-mannanases are grouped by sequence similarity in families GH5, GH26, GH113 and GH134. Family GH113 has been under-explored so far with six enzymes characterized, all from the *Firmicutes* phylum. We undertook the functional characterization of 14 enzymes from a selection of 31 covering the diversity of the family GH113. Our observations suggest that GH113 is a family with specificity towards mannans, with variations in the product profiles and modes of action. We were able to assign mannanase and mannosidase activities to four out of the five clades of the family, increasing by 200% the number of characterized GH113 members, and expanding the toolbox for fine-tuning of mannooligosaccharides.

## Introduction

β-mannans (referred to as mannans throughout this manuscript) define a heterogeneous group of polysaccharides with a common main chain of β-1,4-linked mannopyranoside residues. Mannans are found in the cell wall of algae, in plant seeds and beans, and are a major component of wood hemicelluloses (e.g. galactoglucomannan) embedding the fibril architecture of the matrix [1, 2]. Semi-crystalline mannans participate in the structural architecture of cell walls devoid of cellulose fibrils such as the algae *Porphyra sp*. [3] or *Acetabularia sp* [4–6]. In crystalline mannan, the polysaccharide structure and packing of the chains are very similar to those of cellulose [7]. Mannans can also be extracted from beans or seeds giving gums appreciated in food industry (e.g. guar gum, tara gum, carob gum; [8]). In gums, mannan chains are decorated by carbohydrate components such as galactopyranoside residues in galactomannan gums, or by non-carbohydrate groups (e.g. acetate, sulfate) depending on the botanical origin of the macromolecules. Galactomannan seed gums are already exploited as stabilizer and thickener in the food and cosmetic industries [1, 8]. However, despite their abundance in plant cell wall mannans are under-exploited and their valorization would be key towards sustainable biorefineries. For example, glucomannans are a co-product of the pulp and paper industry, more especially when the process leads to the production of highly refined cellulose (e.g. nanocrystalline cellulose) which requires the extraction of hemicelluloses [9]. Potential applications of wood glucomannan as emulsifiers and stabilizers [10] or platform chemicals [11] have started to be evaluated. Recently, oligo-mannans were found to promote growth of some beneficial bacteria in animal and human gut microbiota, suggesting that these oligosaccharides could be used advantageously as ingredient in prebiotic formulation [12–14]. These investigations revealed that the properties of the series of oligo-mannans were correlated to their size and their degree of substitution. As examples, a degree of polymerization of 2 to 3 (DP2/DP3) had a positive effect on the growth of *Lactobacillus sp*. [15] while DP3/DP4 were more favorable for the growth of *Lactobacillus reuteri* [16]. This example illustrates the need for fine-tuning of the oligo-mannan structures, which requires appropriate enzymes with well-defined recognition properties and mode of action.

The cleavage of β-linked mannopyranose residues is catalyzed by β-mannanases (EC 3.2.1.78) found in glycoside hydrolase families: GH5, GH26, GH113, and GH134 of the Carbohydrate-Active enzyme classification (CAZy, www.cazy.org [17]). GH134 β-mannanases adopt a three -dimensional structure similar to that of lysozyme [18] and cleave the glycosidic bond with an inversion of the anomeric configuration [19]. Contrasting with family GH134, the other families of mannan-degrading enzymes belong to same structural clan GH-A. Accordingly, mannanases from families GH5, GH26 and GH113 share remote homology, notably testified by the same (β/α)_8_ barrel three-dimensional structure, the same catalytic amino acids (Glu/Glu, catalytic nucleophile/catalytic proton donor) and the same catalytic mechanism leading to the retention of the anomeric configuration. GH5 and GH26 are longtime known families, from which numerous members have been characterized, whereas GH113 is a more recent, less studied family. The CAZy website (October 2021) listed for the GH113 family: 1,346 protein sequences from bacterial origin, six sequences from Archaea, four sequences from viruses and nine sequences from Eukaryotic origin, reflecting the bias in taxonomical sequencing. Among Archaea and Eukaryota encoding a GH113, most of the organisms seem to be from aquatic environment. To date, six GH113 enzymes have been biochemically characterized, namely from *Alicyclobacillus acidocaldarius* TC-12-31 [20], *Alicyclobacillus sp*. [21], *Amphibacillus xylanus* NBRC 15112 [22], *Bacillus sp*. N16-5 [23], *Roseburia intestinalis* L1-82 [24] and *Faecalibacterium praunsnitzii* SL3/3 (the latter was published during the course of this study [25]). All these enzymes degrade β-mannan polysaccharides and/or oligosaccharides: endo-β-mannanase activity has been identified for four of them (i.e. the enzymes cleave randomly the mannan backbone), and reducing-end exo-mannosidase activity was described for *R*. *intestinalis* and *F*. *spraunitzii* enzymes (i.e. the enzymes release mannose from the extremity of manno-oligosaccharides). The crystal structures of three GH113 enzymes have been solved, confirming their belonging to clan GH-A [20–22]. Hence, the diversity of GH113 family remains little explored with few enzymes characterized with high sequence similarity (>54% identity on the catalytic domain sequences across enzymes).

In this context, we selected a set of 31 enzymes on the basis of protein sequence diversity, aiming at unveiling the functional diversity of the under-explored GH113 family.

## Results

### Bioinformatic analysis of the GH113 family

**Fig 1:**
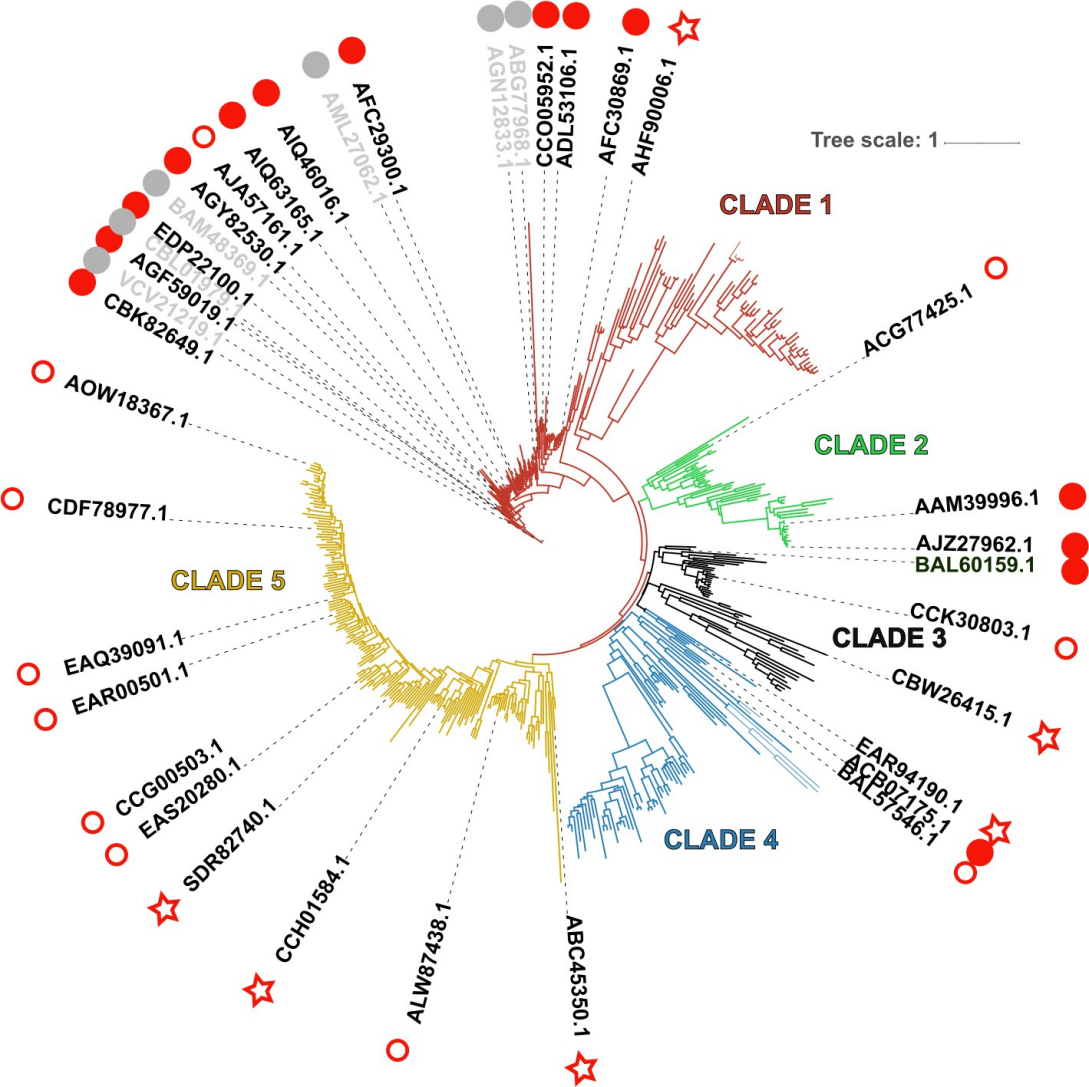
Phylogenetic tree of family GH113. Enzymes studied here are shown with red symbols: filled dots indicate that an activity was assigned to the enzyme, empty ones indicate that no activity could be demonstrated. Stars indicate that the target protein was not obtained in a soluble form. Enzymes characterized in previous studies are labeled in grey. A high-resolution phylogenetic tree is available in Supplementary Material, S1 Fig.

Analysis of the GH113-containing proteins revealed that the vast majority are composed of this sole domain, with a single exception in the marine bacterium *Ardenticatena sp*. whose GH113 bears a Carbohydrate Binding Module (CBM) from family CBM8. Binding to cellulose has been demonstrated for one CBM8 which shares only 36% identity with the CBM8 from *Ardenticatena sp*. Many other CBM8 sequences are found attached to a variety of enzymes, including GH18 enzymes which are mostly chitinases, or, interestingly, putative mannanases from the GH5_40 subfamily. Some sequences carry a C-terminal extension that careful analysis did not allow to relate to any known CBM or any other known module. Signal peptides were identified on approximately 54% of the sequences. A distance tree was built from the catalytic domain sequences of 511 enzymes belonging to GH113 family. This tree showed two large and three small clades driven mostly by the taxonomy (Figs 1 and S1). The large Clade 1 mainly encompasses sequences belonging to the Firmicutes phylum, including all enzymes characterized to date. Most of the sequences in Clade 1 are devoid of signal peptides (83%), suggesting an intra-cellular localization of the enzymes. The other clades are unexplored so far, without any characterized representatives. Clades 2, 3 and 4 are small clades gathering enzymes from various phyla: Clade 2 gathers mostly Alphaproteobacteria and Gammaproteobacteria, Clade 3 Actinobacteria and the few eukaryotic sequences, and Clade 4 other Alphaproteobacteria and a few diverse phyla including Archae. Signal peptides were predicted on 56% for Clade 2, 84% for Clade 3 and 57% for Clade 4. Finally, the large Clade 5 is dominated by Bacteroidetes species, including many strains isolated from marine environment. Signal peptides were frequently encountered in this clade (83%), suggesting an extracellular localization. The phylogenetic tree was used as a guide to select a panel of 31 enzymes, covering the diversity of the GH113 family, for biochemical investigation (Table 1 and Figs 1 and S1).

**Table 1:** List of enzymes studied in this work, their characteristics and qualitative activities on mannan substrates.

Strains	GenBank	SigP	Sol	GlcM	GalM	M	Oligo-M	Mode of action
**Clade 1**								
*Opitutaceae bacterium* TAV5	AHF90006.1	N	-	na	na	na	na	
*Ruminococcus bicirculans*	CCO05952.1	N	+	+	+	+ (M2)	nd	Endo
*Clostridium cellulovorans* 743B	ADL53106.1	N	+	+	+	+ (M2)	nd	Endo
*Paenibacillus mucilaginosus* 3016	AFC30869.1	N	+	+	+	+ (M2)	nd	Endo
*Paenibacillus mucilaginosus* 3016	AFC29300.1	N	+	+	+	+ (M2)	nd	Endo
*Paenibacillus stellifer*	AIQ63165.1	N	+	-	-	-	+ (M2+M1)	Exo
*Paenibacillus sp*. FSL R7-0273	AIQ46016.1	N	+	-	-	-	+ (M2+M1)	Exo
*Carnobacterium sp*. WN1359	AGY82530.1	N	+	-	-	-	+ (M2+M1)	Exo
*Lactococcus lactis subsp*. *Lactis*	AJA57161.1	N	+	-	-	-	-	
*Faecalibacterium prausnitzii* M21/2	EDP22100.1	N	+	-	-	(+)	+ (M2+M1)	Exo
*Coprococcus sp*. ART55/1	CBK82649.1	N	+	+	+	+	nd	Endo
*Clostridium saccharoperbutylacetonicum* N1-4(HMT)	AGF59019.1	N	+	-	-	-	+ (M2+M1)	Exo
*Alicyclobacillus acidocaldarius* TC-12-31 (*Aa*Man113A)*	ABG77968.1							Endo*
*Alicyclobacillus sp*. (Man113A)*	AGN12833.1							Endo*
*Amphibacillus xylanus* NBRC 15112 (*Ax*Man113A)*	BAM48369.1							Endo*
*Bacillus sp*. N16-5 (Man113A)*	AML27062.1							Endo*
*Faecalibacterium prausnitzii* SL3/3	CBL01979.1							Exo*
*Roseburia intestinalis* L1-82 (*Ri*GH113)*	VCV21219.1							Exo*
**Clade 2**								
*Phenylobacterium zucineum* HLK1	ACG77425.1	N	+	-	-	-	-	
*Xanthomonas campestris str*. ATCC 33913	AAM39996.1	Y	+	+	+	+ (M3+M4)	nd	Endo
*Xanthomonas citri subsp*. *citri*	AJZ27962.1	Y	+	+	+	+ (M3+M4)	nd	Endo
**Clade 3**								
*Candidatus Acetothermus autotrophicum*	BAL60159.1	N	+	-	-	+ (M3+M4)	nd	Endo
*Streptomyces davawensis* JCM 4913	CCK30803.1	N	+	-	-	-	-	
*Tetrahymena thermophila* SB210	EAR94190.1	Y	-	na	na	na	na	
*Halobacteriovorax marinus* SJ	CBW26415.1	N	-	na	na	na	na	
**Clade 4**								
*Candidatus Korarchaeum cryptofilum* OPF8	ACB07175.1	Y	+	+	+	+ (M3+M4)	nd	Endo
*uncultured Acetothermia bacterium*	BAL57546.1	Y	+	-	-	-	-	
**Clade 5**								
*Salinibacter ruber* DSM 13855	ABC45350.1	N	-	na	na	na	na	
*Hymenobacter sp*. DG5B	ALW87438.1	N	+	-	-	-	-	
*Gramella sp*. MAR_2010_102	SDR82740.1	N	-	na	na	na	na	
*uncultured Flavobacteriia bacterium*	CCG00503.1	Y	+	-	-	-	-	
*Maribacter sp*. HTCC2170	EAR00501.1	Y	+	-	-	-	-	
*Dokdonia sp*. *M*ED134	EAQ39091.1	Y	+	-	-	-	-	
*Flavobacteria bacterium* BBFL7	EAS20280.1	Y	+	-	-	-	-	
*Polaribacter vadi*	AOW18367.1	Y	+	-	-	-	-	
*Formosa agariphila* KMM 3901	CDF78977.1	Y	+	-	-	-	-	
*Fibrella aestuarina* BUZ 2	CCH01584.1	N	-	na	na	na	na	

Main end-products are given into brackets for linear polysaccharides. SigP: Signal peptide (Y for predicted; N otherwise). Sol: Expression of soluble proteins. Substrates: + for degradation of the substrate; (+) for a weak degradation and—for no degradation. nd: not detected. na: not applicable

*: results from previous works. Underlined GenBank accession numbers highlight sequences for which the 3D structure was solved.

(GlcM: Glucomannan, M: β (1,4) mannan, GalM: galactomannan, OligoM: Oligo-mannans).

### Production of GH113 β-1,4 mannanases and substrate specificity

For this study, codon optimization for heterologous expression in *E*. *coli*, gene synthesis and cloning of the 31 targets were outsourced (Nzytech, Portugal). Signal peptides and other extensions were trimmed in order to conserve only the predicted catalytic domain. Twenty-five targets (81%) were successfully overexpressed in *E*. *coli* BL21(DE3) and soluble. The remaining six proteins were produced as insoluble inclusion body, notably the eukaryotic GH113 from *Tetrahymena thermophila* and an enzyme from the extremely halophilic bacterium *Salinibacter ruber*. The purity of enzymes was attested by a single band visible on SDS-PAGE and estimated to be >90%. The molecular weights were ranging from 35 kDa to 47 kDa, as expected. Final protein concentrations were between approximately 2 and 6 mg.mL^-1^. All the enzymes were produced and screened at least twice to demonstrate reproducibility.

At first, the purified proteins were screened for activity on a set of various substrates, including several mannan polysaccharides from different biological origins (e.g. β-1,4-mannan, glucomannan, galactomannan) (S2 Fig), manno-oligosaccharides and *p*NP-β-mannopyranoside (S1 and S2 Tables). Out of the 25 soluble enzymes, 14 were found to be active on at least one mannan or manno-oligosaccharide substrate. Active enzymes were identified in all Clades but Clade 5, where no member showed even a weak activity. The seven Bacteroides proteins of Clade 5 as well as proteins from other clades for which no activity had been detected on the first set of substrates were subjected to a second screening round on a complementary set of substrates (S2 Table). Despite the diversity of polysaccharides, oligosaccharides and synthetic substrates tested, no activity was observed for any of the proteins, including all the proteins from Clade 5. Qualitative assessment of the degradation capability of the various mannans and manno-oligosaccharides by enzymes from Clades 1 to 4 is given in Table 1.

### Analysis of the mode of action for GH113 β-mannanases on debranched beta -1,4 mannan

**Fig 2:**
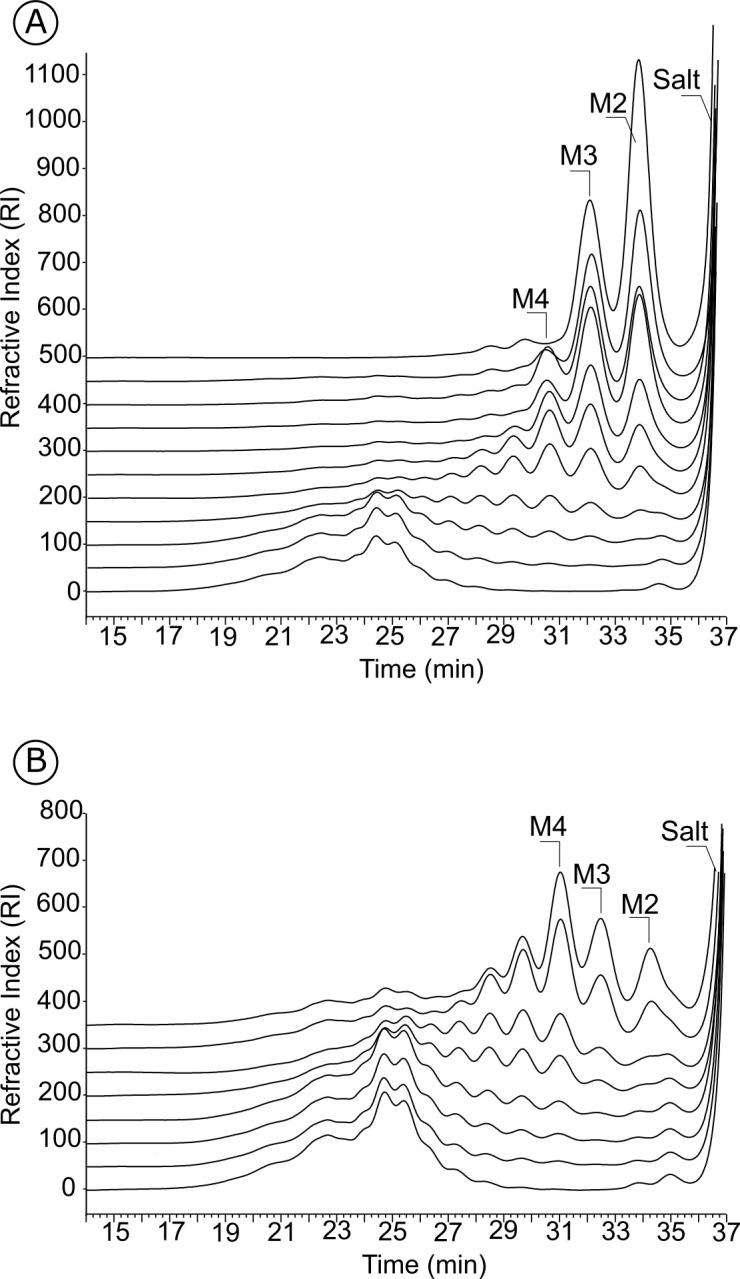
Analysis of the endo mode of action of two GH113 representatives. The degradation of debranched β 1,4 mannan by A: CCO05952 (Clade 1) and B: AJZ27962 (Clade 2) was monitored using size-exclusion chromatography. M2: mannobiose, M3: mannotriose, M4: mannotetraose.

**Fig 3:**
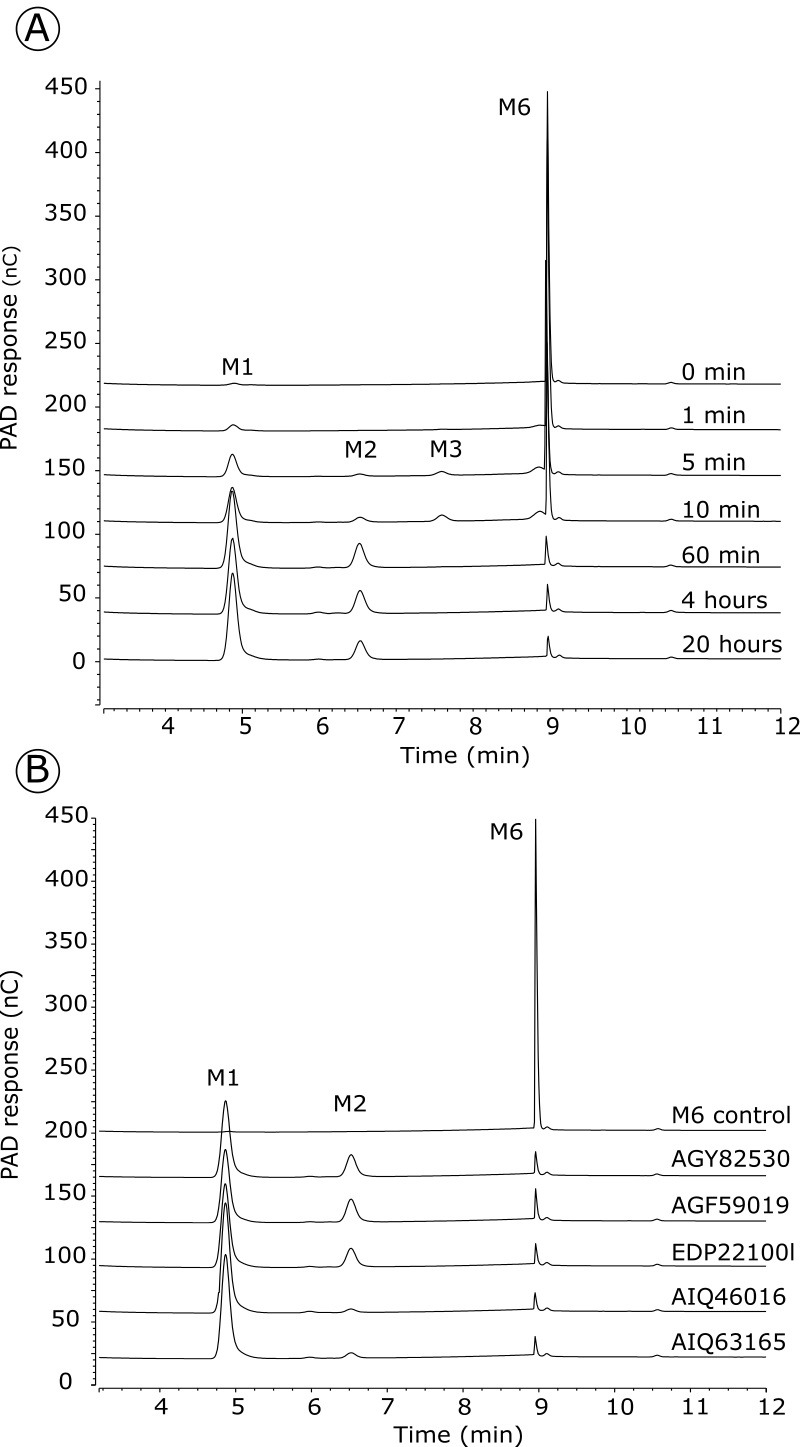
Analysis of the exo mode of action of five GH113 Clade 1 representatives. A: The degradation of mannohexaose (M6) by EDP22100 was monitored by HPAEC-PAD. B: Analysis of end-products released by AGY82530, AGF59019, AIQ46016 and AIQ63165 after overnight incubation with M6. M1: Mannose, M2: mannobiose, M3: mannotriose, M6: mannohexaose.

End-products and mode of action of the enzymes were determined by size-exclusion chromatography. Endo-acting enzymes cleave the polymeric substrate into a range of oligosaccharides whose sizes decrease as the degradation proceeds. Accordingly, five enzymes of Clade 1 revealed an endo-mode of action on linear beta-1,4 mannan (Fig 2). Five other enzymes exhibited activity on a mix of linear manno-oligosaccharides, whereas no significant activity was measured on polysaccharides or on *p*NP-β-mannopyranoside. This is exemplified with EDP22100 (S3 Fig), which accumulated M2 and M1 after extended incubation with oligosaccharides. The activity of EDP22100 was further examined by HPAEC-PAD (Fig 3A) confirming the release of mannose from mannohexaose (M6). Intermediate products were detected in the shorter incubation times and quickly degraded. This analysis confirmed the exo-mannosidase activity that was also described in another *F*. *prausnitzii* strain during the course of our study [25]. To elucidate whether they were mannobiosidases or mannosidases, the four other enzymes with activity on oligosaccharides were similarly subjected to HPAEC-PAD analysis (Fig 3B). All of them were able to release mannose from M6, confirming that they are also exo-mannosidases.

Clade 1 endo-acting enzymes were active on all tested mannans (e.g. glucomannan, galactomannan, beta-1,4-mannan) and released mostly mannobiose (M2) and mannotriose (M3) as main end-products as shown with CCO05952 in Fig 3A. Four enzymes from Clades 2, 3 and 4 were also found to be active on β-mannan polysaccharides. Degradation kinetics monitored by chromatography revealed they were all endo-acting enzymes, releasing a range of oligosaccharides over the course of the reaction. mannotriose (M3) and mannotetraose (M4) were the main end products, as illustrated with the enzyme AJZ27962 on linear mannan (Fig 3B). No transglycosylation activity was observed with any of the tested enzymes in our experimental conditions. Specific activities were determined for one representative from Clades 1 and 2, CCO05952 and AJZ27962, respectively, showing that Clade 1 CCO05952 exhibits a similar activity on various mannans, whereas Clade 2 AJZ27962 has highest activity on glucomannan and decreasing activity with increasing galactose substitutions (Table 2).

**Table 2:** Specific activity of representatives of M1+ M2 producing enzymes (AJZ27962.1, Clade 1) and M3+M4 producing enzymes (CCO05952.1, Clade 2) towards mannans.

	Specific activity (U/mg)	
	AJZ27962.1	CCO05952.1
Konjac glucomannan (0% Gal, mannose:glucose = 60:40)	9.39 +/-0.51	3.00 +/-0.30
Carob linear β-1,4 mannan (0% Gal)	4.73 +/-0.64	3.35 +/-0.0
Carob galactomannan (22% Gal)	4.14 +/-0.45	3.04 +/-0.0
Guar gum (50% Gal)	0.89 +/-0.34	2.79 +/-0.59

## Discussion

### Substrate specificity in the GH113 family

Activity on β-1,4-linked mannose-containing substrates was demonstrated for enzymes grouped in Clades 1 to 4 of the GH113 family. The specificity and the mode of action of these enzymes were carefully determined using a variety of mannan substrates. These analyses revealed different degradation profiles correlated to the mode of action and probably to the active site topology of the enzymes. Five enzymes of Clade 1 were found to be endo-acting enzymes producing mainly M2 and M3 end-products similarly to most of the other previously characterized enzymes grouped in this Clade [20–23]. Five enzymes of Clade 1 had exo-mannosidase activity on oligomannans. All the enzymes grouped in Clade 1 were inactive against *p*NP-β-mannopyranoside, suggesting that productive binding requires at least two sub-sites. Inspection of sequence alignment showed that amino acids involved in the catalytic machinery and interaction with the substrates deduced from crystallographic studies [21, 22] are well conserved in this clade (S4 Fig). In addition, it did not reveal significant differences between endo- and exo-acting enzymes such as, for example, additional loop that could modify the active site topology or absence of specific amino acids that could be involved specially in substrate interactions. This probably explains that exo- and endo-acting enzymes do not group as independent branches in the phylogenetic tree.

To the best of our knowledge, no member of other Clades than the Firmicutes Clade 1 had been studied before the present work. Four out of the seven enzymes selected here among Clades 2, 3 and 4 have been identified as endo-acting mannanases. They show catalytic characteristics different from what is observed for Clade 1. Notably, all of the active enzymes from these three clades exhibit a mode of action leading to the production of longer oligosaccharides than enzymes from Clade 1, mannotriose (M3) and mannotetraose (M4) being the main end-products. One can hypothesize that the active site should be able to accommodate longer substrates and probably presents additional sub-sites to those described in crystallographic studies of Clade 1 mannanases. Accordingly, we notice that enzymes grouped in Clade 1 are generally devoid of signal peptide, whereas enzymes of Clades 2, 3 and 4 more frequently encode one. Therefore, one can hypothesize that the former are in charge of processing internalized mannan oligomers, whereas the latter might target longer polysaccharides outside of the cell. Accordingly, the studies of *R*. *intestinalis Ri*113A [24] and *Bacillus sp*. N16-5 [23] revealed that the GH113-encoding genes were co-localized in the genomes with genes encoding other enzymes involved in the breakdown of glucomannan, galactomannan and galactoglucomannan. Among these enzymes, endo-acting mannanases classified in GH5 or GH26 were identified as the first degraders of the polysaccharide chains. However, this is not supported by an analysis of CAZyme content for Clade 1 species, which revealed that a majority of these species do not encode any other mannan-degrading enzyme from known CAZy family (S5 Fig).

### GH113 of unknown function

Beyond the assignment of mannanase and mannobiosidase activities of Clades 1 to 4, we were not able to identify the specific substrate of selected enzymes grouped in the large Clade 5. Sequence alignments showed that amino acids involved in the substrate recognition as well as those required for the catalytic machinery are well conserved (S4 Fig). Although disappointing, our observations confirm the importance of biochemical characterization to ascribe function of putative enzymes grouped in CAZy families. Challenging bioinformatics predictions with experimental data allows refining annotation methods and highlights the limitations of functional prediction and the attribution of a target substrate. Clade 5 mainly groups enzymes found in Bacteroidetes species living in marine environment. Therefore, the preferred substrate of these enzymes could be a poly- or oligosaccharide occurring in a marine organism but absent of our collection of substrates. This illustrates that functional characterization of CAZymes is often limited by the availability of substrates. Bacteroidetes species are known for displaying a large arsenal of Polysaccharide Utilization Loci (PUL, http://www.cazy.org/PULDB/, [26]), genomic organizations where genes encoding all the activities leading to the complete degradation of a specific polysaccharide (import, cleavage, regulation) are co-localized and co-regulated. Therefore, the occurrence of a gene in a PUL may help to infer its functional role in the catabolism pathway. In the case of the Bacteroidetes GH113 (Clade 5) there is a small number of genes predicted in a PUL compared to the size of the family (26/661, 4%). Moreover, these PULs contain none or a single additional degradative CAZyme (only three *Cyclobacterium* species having two GH144-encoding genes, usually involved in β-1,2 glucan breakdown). This suggests that these enzymes are unlikely to participate in a complex polysaccharide uptake. They might instead be used for the construction and/or remodeling of biofilm or an energy storage polysaccharide in these bacteria, as suggested by their presence on the genome close to Glycosyltransferase (GT) genes of family GT2, encoding enzymes involved in polysaccharide synthesis. Accordingly, we observed that among the large diversity of specificities in family GT2, some of the GT2 located in GH113-containing PULs exhibit similarity with mannosyltransferases.

## Conclusion

The rational exploration of the diversity of GH113 family revealed that this family is probably a family targeting specifically mannans. The phylogenetic analysis suggested that all six members characterized so far belong to a unique clade mostly restricted to the Firmicutes phylum for which we further characterized six additional members. We further investigated three other clades without characterized members so far, that cover a broader taxonomic diversity, and we demonstrated the mannanase activity of GH113 from two Proteobacteria, an unclassified Bacteria and an Archeae. In a large fifth clade, specific of the Bacteroidetes phylum, despite the conservation of key amino acids, we were unable to ascribe a function to these proteins or even confirm that they are glycoside hydrolases.

This study increases by 200% the number of characterized GH113 and, interestingly, we observed different modes of action (e.g. endo-/exo) and various modalities of substrate recognition leading to different end-products. The set of enzymes studied herein complete the enzymes toolbox for the preparation of series oligo-mannans that may find some application, for example to produce prebiotics.

## Materials and methods

### Bioinformatics analyses and sequence selection

Protein accessions of non-fragmentary GH113 enzymes were extracted from the CAZy database (May 2021) and used to retrieve the corresponding amino-acid sequences from the NCBI database. The amino acid sequences were trimmed to isolate the GH113 catalytic domain. To reduce the initial set of 1319 sequences, we performed a CD-HIT [27] with a sequence identity threshold of 95%. The resulting 511 sequences were aligned using MAFFT tool (version 7.453, [28]) following the accuracy-oriented method with the option maxiterate of 1000. TrimAl v1.2 [29] was used to automatically remove poorly aligned regions from the alignment with the automated option. The alignment was used to compute a distance matrix based on maximum likelihood distances [30]. The resulting matrix distance was then used to construct a phylogenetic tree using FastME [31]. Peptide signals were predicted using Phobius [32].

### Synthesis of GH113 encoding genes

The selected GH113 sequences are listed in Table 1. After removal of the signal peptide sequence when present and codon optimization for expression in *Escherichia coli*, the sequences were synthesized and inserted in pHTP1 expression vector in frame with a (His)_6_-tag located at the N-terminal end of the proteins (NZyTech, Portugal).

### Heterologous expression and purification of recombinant GH113

Approximately 20 ng of each plasmid were used to transform *E*. *coli* BL21(DE3) competent cells. Resulting clones harboring the recombinant expression plasmids were grown in 5 ml LB precultures supplemented with 30 μg/mL kanamycin in a shaking incubator at 180 rpm and 37°C overnight. Cultures were subsequently carried out in 50 ml of NZY auto-induction LB media (NZYtech) medium supplemented with 30 μg/mL kanamycin, with 1/100 v/v overnight precultures and further incubated at 25°C for 30 hrs. Cultures were stopped by centrifugation at 4000*g* for 5 min. The bacterial pellet was resuspended in buffer A (50 mm Tris pH7.8, 300 mM NaCl, 10 mM Imidazole) with 50 μg/ml lysozyme and stored at -80°C overnight. After thawing, resuspended cells were incubated with DNAse at 10°C for 15 min. Insoluble fractions were removed by centrifugation at 40,000*g* for 30 min at 4°C. The proteins were purified by affinity chromatography using a nickel agarose affinity resin (Ni-NTA resin, Qiagen) loaded on poly-prep® chromatography columns (Bio-Rad), as described in [33]. The resin was equilibrated with buffer A and the His_6_-tagged recombinant enzymes were eluted with buffer A containing 300 mM imidazole. The purity of the fractions was estimated by 10% SDS-PAGE analysis. Before biochemical characterization, selected proteins were further purified on a gel permeation ENRich650 column (Bio-Rad) and eluted in 20 mM Tris-HCl pH 7.8, 100 mM NaCl. The purity of the fractions was assessed using 10% SDS-PAGE analysis.

### Enzyme activity screening

#### Synthetic substrate assay

The list of *p*-nitrophenyl substrates (Sigma Aldrich) used in this study is given in S2 Table. Activities toward 5 mM pNP substrates were determined by measuring the release of 4-nitrophenol in 100 mM Tris HCl pH 7.8, NaCl 50 mM, using a 200-μl reaction volume and suitably diluted enzymes. Reactions mixtures were incubated at room temperature for 2 to 16 hrs and the release of 4-nitrophenol was quantified at 405 nm.

#### Polysaccharide assay

Polysaccharide originating from plant cell wall, seeds, animal and marine environment, were screened. The list of polysaccharide substrates tested in this study is available in S1 Table. Substrates were prepared as 0.4% solutions or suspensions in the case of insoluble substrates (i.e. ivory nut mannan, carob debranched mannan). To identify GH113 enzyme substrates, 200 μl reactions containing 0.2% substrate and suitably diluted enzyme (0.4–10.9 μM per assay) in 100 mM Tris HCl pH7.8, 50 mM NaCl were incubated in 10 kDa-microfiltration plates (10 kDa, PES, Pall corporation) for 16 hrs at 25°C under shaking. The reaction was terminated by filtration of reaction mixtures on a multiscreen HTS vacuum manifold (MSVMHTS00, Millipore) connected to a high-output vacuum pressure pump (Millipore). The filtrates were then analyzed by assaying reducing sugars using the ferricyanide assay [34] as described before [35].

#### Oligosaccharide assay

Oligosaccharides were prepared in-house from linear β-1,4 mannan. Ten milliliters of 0.4% mannan suspension were incubated with 100 μg of GH113 (AJZ27962.1) in Tris HCl 100 mM pH 7.8, NaCl 50 mM for 30 hrs at 25°C. The reaction was terminated by boiling for 10 min. After filtration on a 0.22 μm PES membrane, the obtained oligosaccharides were stored at -20°C until use. GH113 activities toward oligosaccharides were determined as follows: 100 μl of oligosaccharide solution were incubated with suitably diluted enzymes in Tris HCl 100 mM pH7.8, 50 mM NaCl and incubated for 16 hrs at 25°C under shaking, as described previously. The reactions were terminated by boiling for 5 min and products were analyzed as described in the next section. For M6 degradation experiments, 100μM M6 were incubated with the suitably diluted enzymes in 10 mM phosphate buffer pH 7.5 at 25°C under shaking and the reaction was terminated by boiling for 10 min and filtered on 10 kDa cut-off PES membranes.

### Gel permeation chromatography

Enzymatic degradation of polysaccharides and oligosaccharides was validated by gel permeation chromatography using a Superdex peptide 10/300 (GE Healthcare) column connected to a high-performance liquid chromatography (HPLC) Ultimate 3000 system (Thermo Fisher). Injection volume was 50 μL and the elution was performed at 0.5 ml.min^-1^ in 0.1 M NaCl. Oligosaccharides were detected by differential refractometry (Iota 2 differential refractive index detector, Precision Instruments). Mannose and mannooligosaccharides ranging from DP2 to DP4 were used as standards.

### HPAEC-PAD

Enzymatic degradation of M6 was followed using high-performance anion-exchange chromatography with pulsed amperometric detection on a Dionex ICS-6000 system. Hydrolysis products were loaded onto a CarboPac PA100 2 x 250 mm column coupled to a CarboPac PA100 2 x 50 mm guard column. Injection volume was 5 μl and flow rate was 0.25 ml/min. Elution was carried out from 0 to 15 min in NaOH 0.1 M with a gradient from 0 to 0.5 M sodium acetate. The elution was followed by cleaning and regeneration of the column with 10 min of NaOH 0.2 M and 1M sodium acetate, 10 min NaOH 0.2M and 10 min NaOH 0.1 M.

## Supporting information

S1 Fig High resolution phylogenetic tree of family GH113. Major taxonomic groups were color-coded (tree branches and leaf labels): red for Firmicutes, blue for Alphaproteobacteria, green for Gammaproteobacteria, black for Actinobacteria and brown for Bacteroidetes.
(PNG)

S2 Fig Characteristics of the mannans used in this study. Symbolic representation of glycans is given with blue dots: glucose, green dots: mannose, yellow dots: galactose.
(DOCX)

S3 Fig Degradation of linear mannooligosaccharides by a clade 1 GH113 representative. The degradation of manno-oligosaccharides by EDP22100 was monitored using size-exclusion chromatography. M1: Mannose, M2: Mannobiose, M3: mannotriose, M4: mannotetraose.
(DOCX)

S4 Fig Multiple alignment of selected GH113 sequences. Conserved amino acids are highlighted with a red background, catalytic amino acids are indicated by a grey dot. Multiple alignment was generated using the MEGA Software (Tamura et al., 2021) and the figure was edited using ESPript 3 (Robert and Gouet, 2014).
Supplementary references
Tamura K, Stecher G, Kumar S. MEGA11: molecular evolutionary genetics analysis version 11. Mol Biol Evol. 2021;38:3022–3027. doi: 10.1093/molbev/msab120.
Robert X, Gouet P. Deciphering key features in protein structures with the new ENDscript server. Nucl Acids Res. 2014; 42(W1): W320-W324. doi: 10.1093/nar/gku316).
(DOCX)

S5 Fig Comparison of mannanase-encoding gene repertoires in Clade 1 organisms using double hierarchical clustering. Top tree shows mannanase families or subfamilies, left tree highlights clusters in Clade 1 fungi defined by number of mannanase-encoding genes. Abundance of the different genes within a family is represented by a colour scale from 0 (white) to the maximal number for each family (red) per species. The figure was edited using Morpheus (https://software.broadinstitute.org/morpheus/). A hierarchical clustering was performed with the option "one minus pearson correlation " on the rows. The linkage method used was average.
(DOCX)

S1 Table List of polysaccharides used in this study. CM: Carboxymethyl, CWP: Cell wall polysaccharides.
(DOCX)

S2 Table List of synthetic substrates used in this study. (DOCX)

## Abbreviations

CAZymes: Carbohydrate-Active enZymes
DP: degree of polymerization
GH: Glycoside Hydrolase
HPAEC-PAD: High-performance anion-exchange chromatography with pulsed amperometric detection
M1: Mannose
M2: mannobiose
M3: mannotriose
M4: mannotetraose
M6: Mannohexaose

## References

[1] Rodriguez-Gacio MDC, Iglesias-Fernandez R, Carbonero P, Matilla AJ. Softening-up mannan-rich cell walls. J Exp Bot. 2012;63: 3976–3988. doi: 10.1093/jxb/ers096 22553284

[2] Terrett OM, Lyczakowski JJ, Yu L, Iuga D, Franks WT, Brown SP, et al. Molecular architecture of softwood revealed by solid-state NMR. Nat Commun. 2020;11: 5773. doi: 10.1038/s41467-020-19585-0 31673042 PMC6823442

[3] Frei E, Preston RD. Non-cellulosic structural polysaccharides in algal cell walls—II. association of xylan and mannan in *Porphyra umbilicalis*. Proc R Soc Lond B Biol Sci. 1964;160: 314–327. doi: 10.1098/rspb.1964.0042 14213765

[4] Frei E, Preston RD. Non-cellulosic structural polysaccharides in algal cell walls—III. Mannan in siphoneous green algae. Proc R Soc Lond B Biol Sci. 1968;169: 127–145. doi: 10.1098/rspb.1968.0001 14213764

[5] Chanzy H, Grosrenaud A, Vuong R, Mackie W. The crystalline polymorphism of mannan in plant cell walls and after recrystallisation. Planta. 1984;161: 320–329. doi: 10.1007/BF00398722 24253721

[6] Dunn EK, Shoue DA, Huang XM, Kline RE, MacKay AL, Carpita NC, et al. Spectroscopic and biochemical analysis of regions of the cell wall of the unicellular ’mannan weed’, *Acetabularia acetabulum*. Plant Cell Physiol. 2007;48: 122–133. doi: 10.1093/pcp/pcl053 17169920

[7] Chanzy H, Perez S, Miller DP, Paradossi G, Winter WT. An electron diffraction study of the mannan I crystal and molecular structure. Macromolecules. 1987;20: 2407–2413. doi: 10.1021/ma00176a014

[8] Dierckx S, Dewettinck K. Seed Gums. In: Vandamme E, De Baets S, Steinbüchel A, editors. Biopolymers Vol 6 polysaccharides II: polysaccharides of eukaryotes. Berlin, Wiley VCH; 2002. pp. 321–344.

[9] Rajan K, Djioleu A, Kandhola G, Labbe N, Sakon J, Carrier DJ, et al. Investigating the effects of hemicellulose pre-extraction on the production and characterization of loblolly pine nanocellulose. Cellulose. 2020;27: 3693–3706.

[10] Valoppi F, Lahtinen M, Bhattarai M, Kirjoranta SJ, Juntti VK, Peltonen L, et al. KS. Centrifugal fractionation of softwood extracts improves biorefinery workflow and yields functional emulsifiers. Green Chem. 2019;21: 4691–4705. doi: 10.1039/C9GC02007A

[11] López M, Vila C, Santos V, Parajó JC. Polymers manufacture of platform chemicals from pine wood polysaccharides in media containing acidic ionic liquids. Polymers (Basel). 2020;12(6): 1215. doi: 10.3390/polym12061215 32471027 PMC7362180

[12] La Rosa SL, Kachrimanidou V, Buffetto F, Pope PB, Pudlo NA, Martens EC, et al. Wood-derived dietary fibers promote beneficial human gut microbiota. mSphere. 2019;4: e00554–18. doi: 10.1128/mSphere.00554-18 30674645 PMC6344601

[13] Michalak L, Gaby JC, Lagos L, La Rosa SL, Hvidsten TR, Tetard-Jones C, et al. Microbiota-directed fibre activates both targeted and secondary metabolic shifts in the distal gut. Nat Commun. 2020;11: 5773. doi: 10.1038/s41467-020-19585-0 33188211 PMC7666174

[14] Deloule V, Boisset C, Hannani D, Suau A, Le Gouellec A, Chroboczek J, et al. Prebiotic role of softwood hemicellulose in healthy mice model. J Funct Foods. 2020;64: 103688. doi: 10.1016/j.jff.2019.103688

[15] Mary PR, Prashanth KVH, Vasu P, Kapoor M. Structural diversity and prebiotic potential of short chain β-manno-oligosaccharides generated from guar gum by endo-β-mannanase (ManB-1601). Carbohyd Res. 2019;486: 107822. doi: 10.1016/j.carres.2019.107822 31600611

[16] Kalidas NR, Saminathan M, Ismail IS, Abas F, Maity P, Islam SS, et al. Structural characterization and evaluation of prebiotic activity of oil palm kernel cake mannan oligosaccharides. Food Chem. 2017; 234: 348–355. doi: 10.1016/j.foodchem.2017.04.159 28551246

[17] Drula E, Garron ML, Dogan S, Lombard V, Henrissat B, Terrapon N. The carbohydrate-active enzyme database: functions and literature. Nucleic Acids Res. 2022;50(D1): D571–7. doi: 10.1093/nar/gkab1045 34850161 PMC8728194

[18] Jin Y, Petricevic M, John A, Raich L, Jenkins H, Portela De Souza L, et al. A β-mannanase with a lysozyme-like fold and a novel molecular catalytic mechanism. ACS Cent Sci. 2016;2: 896–903. doi: 10.1021/acscentsci.6b00232 28058278 PMC5200933

[19] Shimizu M, Kaneko Y, Ishihara S, Mochizuki M, Sakai K, Yamada M, et al. Novel β-1,4-mannanase belonging to a new glycoside hydrolase family in *Aspergillus nidulans*. J Biol Chem. 2015;290: 27914–27927. doi: 10.1074/jbc.M115.661645 26385921 PMC4646033

[20] Zhang Y, Ju J, Peng H, Gao F, Zhou C, Zeng Y, et al. Biochemical and structural characterization of the intracellular mannanase *Aa*ManA of *Alicyclobacillus acidocaldarius* reveals a novel glycoside hydrolase family belonging to clan GH-A. J Biol Chem. 2008; 283: 31551–31558. doi: 10.1074/jbc.M803409200 18755688

[21] Xia W, Lu H, Xia M, Cui Y, Bai Y, Qian L, et al. A novel glycoside hydrolase family 113 endo-β-1,4-mannanase from *Alicyclobacillus* sp. strain A4 and insight into the substrate recognition and catalytic mechanism of this family. Appl Environ Microbiol. 2016;82: 2718–2727. doi: 10.1128/AEM.04071-15 26921423 PMC4836435

[22] You X, Qin Z, Yan Q, Yang S, Li Y1, Jiang Z. Structural insights into the catalytic mechanism of a novel glycoside hydrolase family 113 β-1,4-mannanase from *Amphibacillus xylanus*. J Biol Chem. 2018;293(30):11746–11757. doi: 10.1074/jbc.RA118.002363 29871927 PMC6066303

[23] Song Y, Sun W, Fan Y, Xue Y, Liu D, Ma C, et al. Galactomannan degrading enzymes from the mannan utilization gene clade of alkaliphilic *Bacillus sp*. N16-5 and their synergy on galactomannan degradation. J Agric Food Chem. 2018;66: 11055–11063. doi: 10.1021/acs.jafc.8b03878 30351049

[24] La Rosa SL, Leth ML, Michalak L, Hansen ME, Pudlo NA, Glowacki R, et al. The human gut Firmicute *Roseburia intestinalis* is a primary degrader of dietary beta-mannans. Nat Commun. 2019;10: 905. doi: 10.1038/s41467-019-08812-y 30796211 PMC6385246

[25] Lindstadt LJ, Lo G, Leivers S, Lu Z, Michalak L, Pereira GV, et al. Human gut *Faecalibacterium prausnitzii* deploys a highly efficient conserved system to cross-feed on β-mannan-derived oligosaccharides. mBio. 2021;12(3):e0362820. doi: 10.1128/mBio.03628-20 34061597 PMC8262883

[26] Terrapon N, Lombard V, Drula É, Lapébie P, Al-Masaudi S, Gilbert HJ, et al. PULDB: the expanded database of Polysaccharide Utilization Loci. Nucleic Acids Res. 2018;46(D1): D677–D683. doi: 10.1093/nar/gkx1022 29088389 PMC5753385

[27] Li W, Godzik A. Cd-hit: a fast program for clustering and comparing large sets of protein or nucleotide sequences. Bioinformatics. 2006;22(13): 1658–1659. doi: 10.1093/bioinformatics/btl158 16731699

[28] Katoh K, Rozewicki J, Yamada KD. MAFFT online service: multiple sequence alignment, interactive sequence choice and visualization. Brief Bioinform. 2019;20(4): 1160–1166. doi: 10.1093/bib/bbx108 28968734 PMC6781576

[29] Capella-Gutierrez S, Silla-Martinez JM, Gabaldon T. trimAl: a tool for automated alignment trimming. Bioinformatics. 2009;25(15): 1972–1973. doi: 10.1093/bioinformatics/btp348 19505945 PMC2712344

[30] Guindon S, Gascuel O. A simple, fast, and accurate algorithm to estimate large phylogenies by maximum likelihood. Syst Biol. 2003;52: 696–704. doi: 10.1080/10635150390235520 14530136

[31] Lefort V, Desper R, Gascuel 0. FastME 2.0: A comprehensive, accurate, and fast distance-based phylogeny inference program. Mol Biol Evol. 2015;32: 2798–2800. doi: 10.1093/molbev/msv150 26130081 PMC4576710

[32] Käll L, Krogh A, Sonnhammer EL. A combined transmembrane topology and signal peptide prediction method. J Mol Biol. 2004;338: 1027–1036. doi: 10.1016/j.jmb.2004.03.016 15111065

[33] Helbert W, Poulet L, Drouillard S, Mathieu S, Loiodice M, Couturier M, et al. Discovery of novel carbohydrate-active enzymes through the rational exploration of the protein sequences space. Proc Nat Acad Sci U S A. 2019;116:v6063–6068. doi: 10.1073/pnas.1815791116 30850540 PMC6442616

[34] Kidby DK, Davidson DJ. A convenient ferricyanide estimation of reducing sugars in the nanomole range. Anal Biochem. 1973;55:321–325. doi: 10.1016/0003-2697(73)90323-0 4753157

[35] Mathieu S, Henrissat B, Labre F, Skjåk-Bræk G, Helbert W. Functional exploration of the polysaccharide lyase family PL6. PLoS One. 2016;11(7):e0159415. doi: 10.1371/journal.pone.0159415 27438604 PMC4954714

