# Supplementary figures and images for: Functional exploration of the glycoside hydrolase family GH113
Source: PLoS One. 2022 Apr 22;17(4):e0267509. doi: 10.1371/journal.pone.0267509 (PMC9032380; doi:10.1371/journal.pone.0267509)

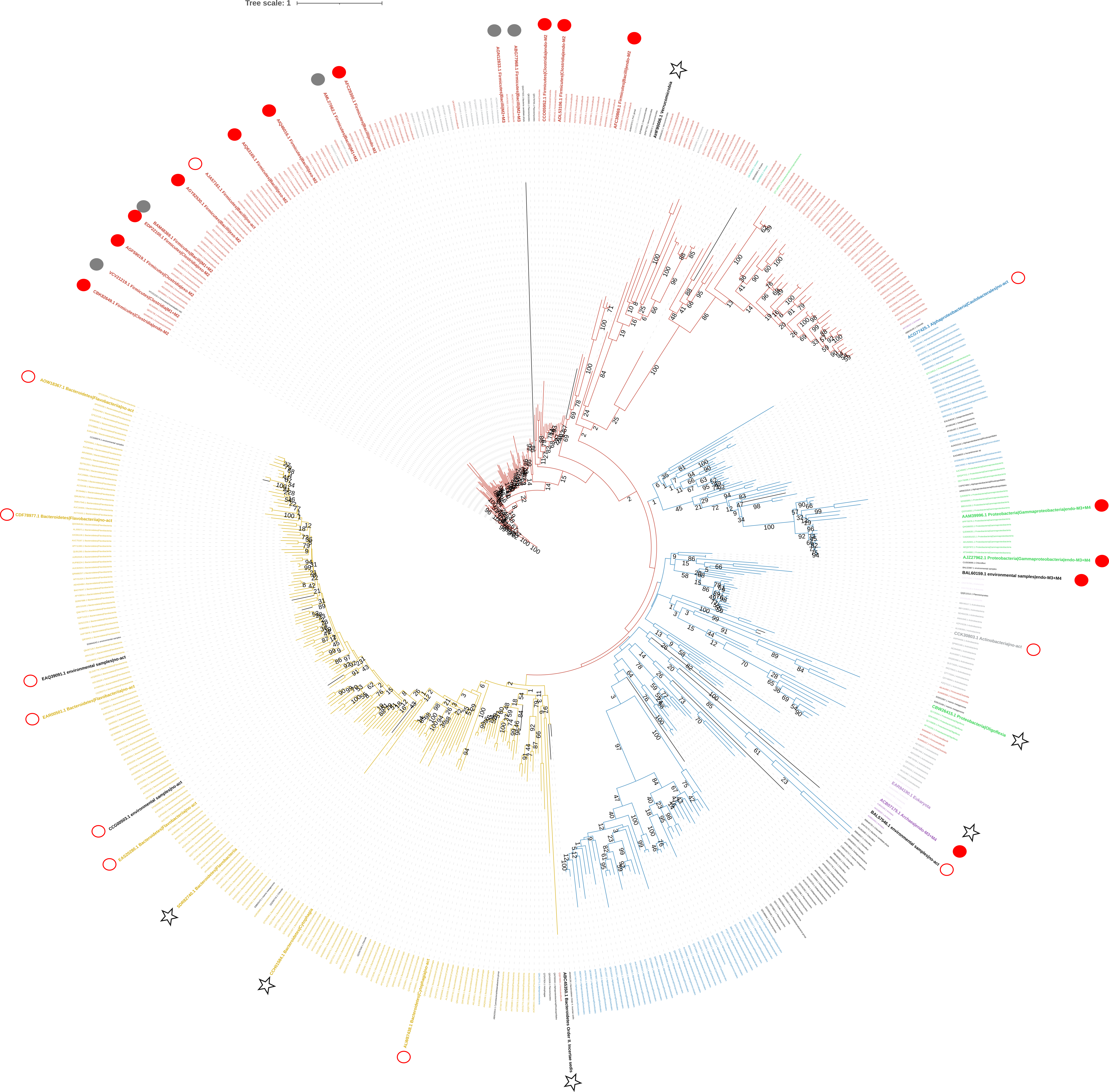

Supplement: S1 Fig — Major taxonomic groups were color-coded (tree branches and leaf labels): red for Firmicutes, blue for Alphaproteobacteria, green for Gammaproteobacteria, black for Actinobacteria and brown for Bacteroidetes. (PNG) [file pone.0267509.s001.png]
