# Supplementary material for: Functional exploration of the glycoside hydrolase family GH113
Source: PLoS One. 2022 Apr 22;17(4):e0267509. doi: 10.1371/journal.pone.0267509 (PMC9032380; doi:10.1371/journal.pone.0267509)
Supplement: S2 Fig — Symbolic representation of glycans is given with blue dots: glucose, green dots: mannose, yellow dots: galactose. (DOCX) [file pone.0267509.s002.docx]

**
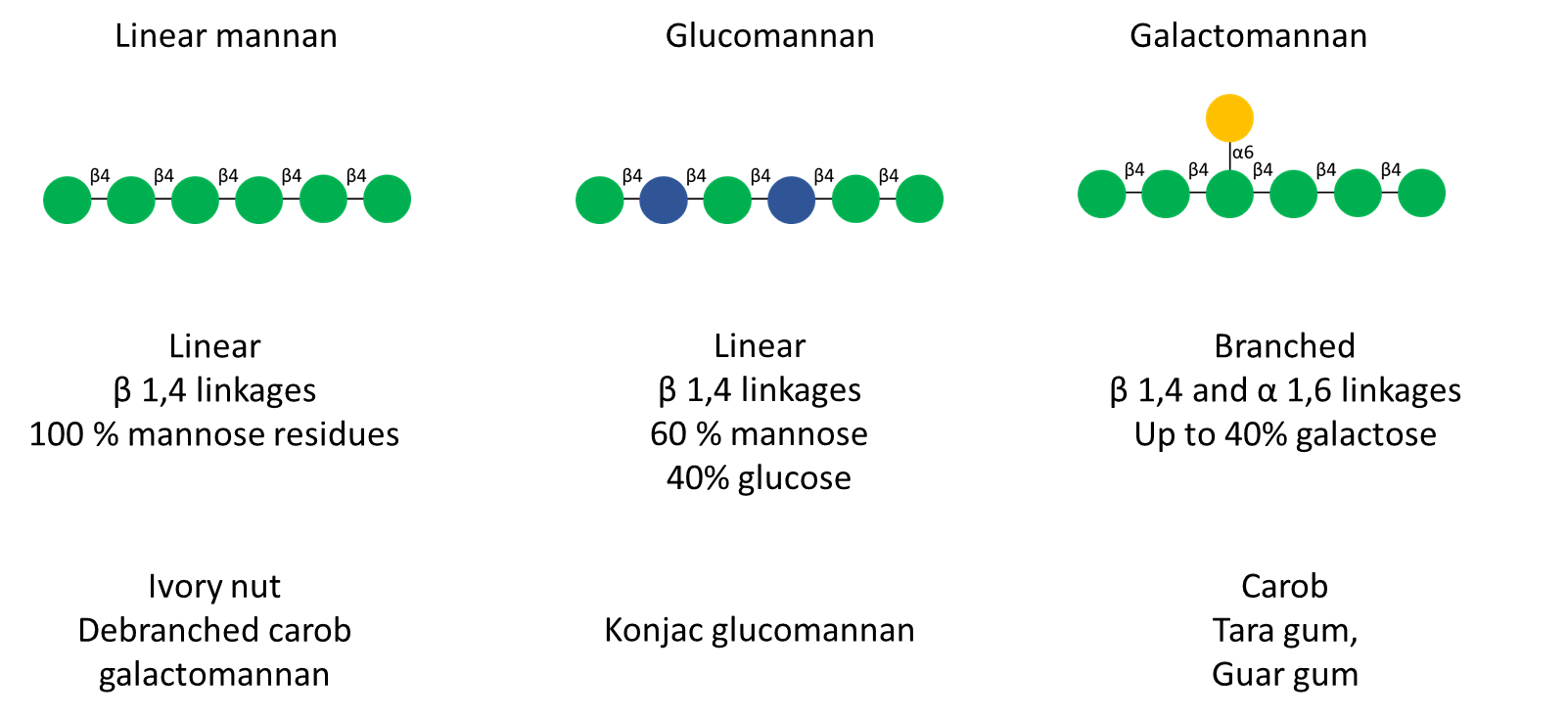
**

**S2 Fig: Characteristics of the mannans used in this study.** Symbolic representation of glycans is given with blue dots: glucose, green dots: mannose, yellow dots: galactose.
