# Supplementary material for: Functional exploration of the glycoside hydrolase family GH113
Source: PLoS One. 2022 Apr 22;17(4):e0267509. doi: 10.1371/journal.pone.0267509 (PMC9032380; doi:10.1371/journal.pone.0267509)
Supplement: S3 Fig — The degradation of manno-oligosaccharides by EDP22100 was monitored using size-exclusion chromatography. M1: Mannose, M2: Mannobiose, M3: mannotriose, M4: mannotetraose. (DOCX) [file pone.0267509.s003.docx]

**
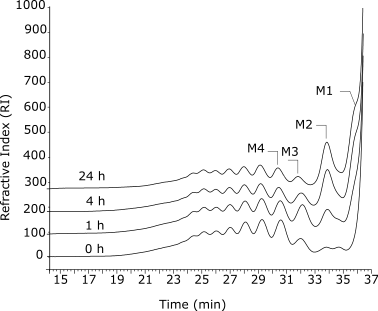
**

**S3 Fig:** **Degradation of linear mannooligosaccharides by a clade 1 GH113 exo-mannosidase representative.** The degradation of manno-oligosaccharides by EDP22100 was monitored using size-exclusion chromatography. M1: Mannose, M2: Mannobiose, M3: mannotriose, M4: mannotetraose
