# Supplementary material for: Functional exploration of the glycoside hydrolase family GH113
Source: PLoS One. 2022 Apr 22;17(4):e0267509. doi: 10.1371/journal.pone.0267509 (PMC9032380; doi:10.1371/journal.pone.0267509)
Supplement: S4 Fig — Conserved amino acids are highlighted with a red background, catalytic amino acids are indicated by a grey dot. Multiple alignment was generated using the MEGA Software (Tamura et al., 2021) and the figure was edited using ESPript 3 (Robert and Gouet, 2014). Supplementary references Tamura K, Stecher G, Kumar S. MEGA11: molecular evolutionary genetics analysis version 11. Mol Biol Evol. 2021;38:3022–3027. doi: 10.1093/molbev/msab120. Robert X, Gouet P. Deciphering key features in protein structures with the new ENDscript server. Nucl Acids Res. 2014; 42(W1): W320-W324. doi: 10.1093/nar/gku316). (DOCX) [file pone.0267509.s004.docx]

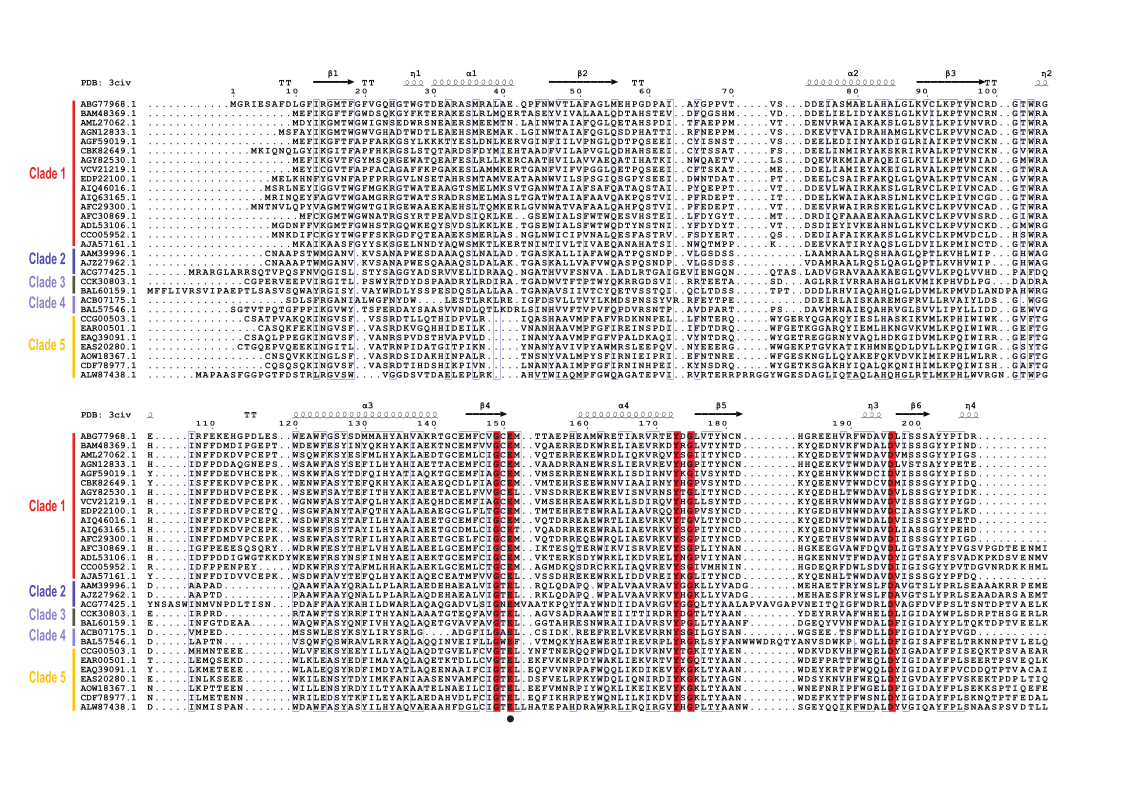

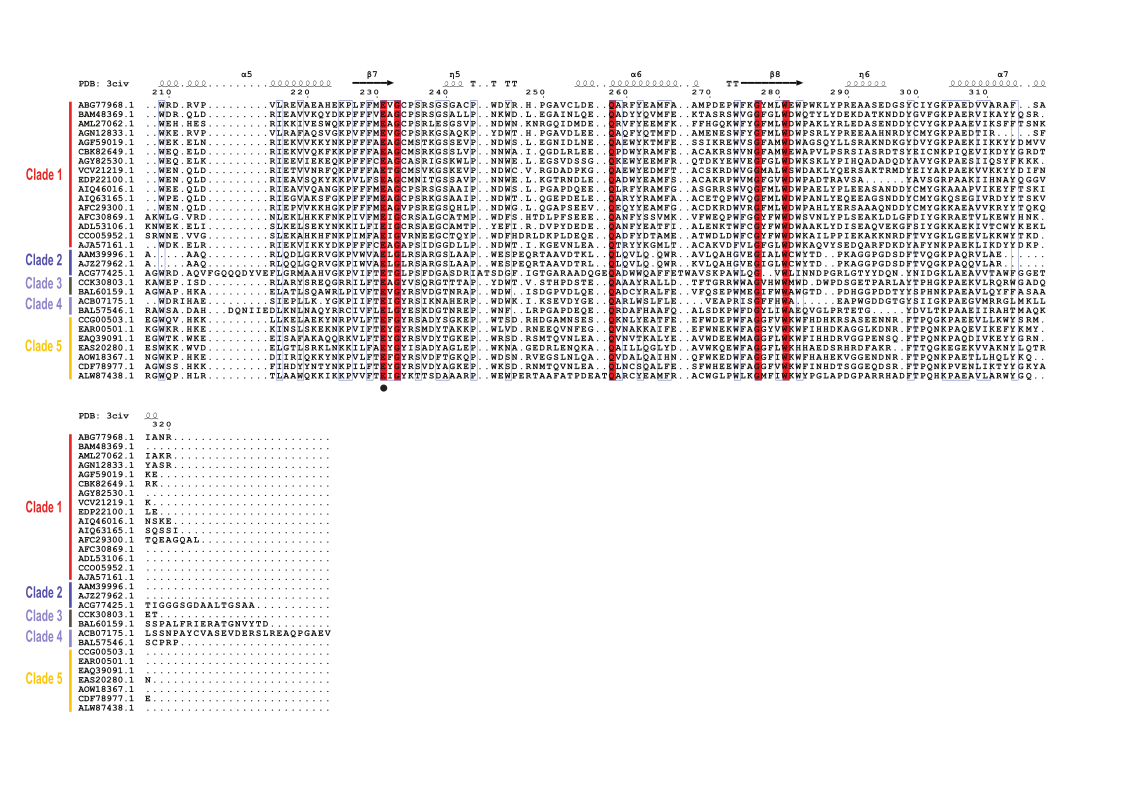


**Fig S4. Multiple alignment of selected GH113 sequences**

Conserved amino acids are highlighted with a red background, catalytic amino acids are indicated by a grey dot. Multiple alignement was generated using the MEGA Software (Tamura et al., 2021) and the figure was edited using ESPript 3 (Robert and Gouet, 2014).
