# Supplementary material for: Functional exploration of the glycoside hydrolase family GH113
Source: PLoS One. 2022 Apr 22;17(4):e0267509. doi: 10.1371/journal.pone.0267509 (PMC9032380; doi:10.1371/journal.pone.0267509)
Supplement: S5 Fig — Top tree shows mannanase families or subfamilies, left tree highlights clusters in Clade 1 fungi defined by number of mannanase-encoding genes. Abundance of the different genes within a family is represented by a colour scale from 0 (white) to the maximal number for each family (red) per species. The figure was edited using Morpheus (https://software.broadinstitute.org/morpheus/). A hierarchical clustering was performed with the option "one minus pearson correlation " on the rows. The linkage method used was average. (DOCX) [file pone.0267509.s005.docx]

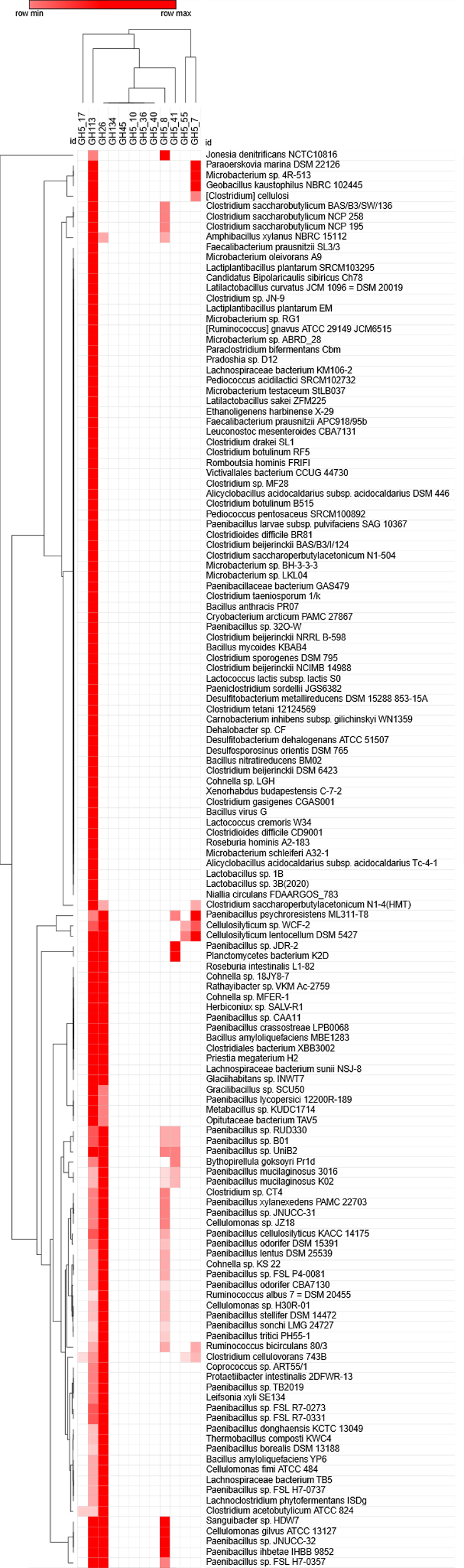


**Fig S5: Comparison of mannanase-encoding gene repertoires in Clade 1 organisms using double hierchical clustering.**

Top tree shows mannanase families or subfamilies, left tree highlights clusters in Clade 1 fungi defined by number of mannanase-encoding genes. Abundance of the different genes within a family is represented by a colour scale from 0 (white) to the maximal number for each family (red) per species. The figure was edited using Morpheus (<https://software.broadinstitute.org/morpheus/>). A Hierarchical Clustering was performed with the option "one minus pearson correlation " on the rows. The linkage method used was average.
