# Supplementary material for: Functional exploration of the glycoside hydrolase family GH113
Source: PLoS One. 2022 Apr 22;17(4):e0267509. doi: 10.1371/journal.pone.0267509 (PMC9032380; doi:10.1371/journal.pone.0267509)
Supplement: S1 Table — CM: Carboxymethyl, CWP: Cell wall polysaccharides. (DOCX) [file pone.0267509.s006.docx]

**S1 Table: List of polysaccharides used in this study.**

|  | **Substrate** | | **Organism / biological source** | | **Provider** | | |
| --- | --- | --- | --- | --- | --- | --- | --- |
| **First round screening** | |  | |  | | |  |
|  | Agarose | |  | | Sigma-Aldrich | | |
|  | Arabic gum | | *Acacia sp.* | | Dextra | | |
|  | Arabinan | | Sugar beet | | Megazyme | | |
|  | Arabinoxylan | | Wheat | | Megazyme | | |
|  | β-1,3 xylan | | *Penicillus dumetosus* | | Prepared in house | | |
|  | β-1,4 mannan (insoluble) | | Ivory nut | | Megazyme | | |
|  | β-1,4 mannan (insoluble) | | Carob | | Megazyme | | |
|  | β-1,4 manno-oligosaccharides | | Hydrolysed carob β-1,4 mannan | | Prepared in house | | |
|  | β-1,4 xylan | | Beechwood | | Sigma-Aldrich | | |
|  | β-glucan | | Barley | | Megazyme | | |
|  | β-glucan | | Oat | | Megazyme | | |
|  | Cellulose (Avicel) | | Cotton | | Sigma-Aldrich | | |
|  | Chitosan | |  | | Dextra | | |
|  | CM-Cellulose | |  | | Sigma-Aldrich | | |
|  | CM-curdlan | |  | | Megazyme | | |
|  | CM-Pachyman | |  | | Megazyme | | |
|  | Curdlan | | *Alcaligenes faecalis* var. myxogenes | | Dextra |  |  |
|  | Galactan | | Potato | | Megazyme |  |  |
|  | Galactomannan | | Carob (*Ceretonia siliqua*) | | Megazyme | | |
|  | Galactomannan | | Guar gum *(Cyamopsis tetragonolobus)* | | Dextra | | |
|  |  | |  | |  | | |
|  | Galactomannan | | Tara gum (*Caesalpina spinosa)* | |  | | |
|  | Glucomannan | | *Amorphophallus konjac* | | Megazyme | | |
|  | Heparin sodium | |  | | Dextra | | |
|  | Hyaluronic acid | | Rooster comb | | Glycomix | | |
|  | Inulin | | Chicory roots | | Megazyme | | |
|  | ι/ν-carrageenan | | *Eucheuma denticulatum* | | Cargill | | |
|  | κ/μ- carrageenan | | *Kappaphycus alvarezii* | | Cargill | | |
|  | Methyl-Pachyman | | *Poria cocos* | | Megazyme | | |
|  | Pachyman | | *Poria cocos* | | Megazyme | | |
|  | Phosphoric acid swollen cellulose | | *Wood avicel* | | Helbert et al, 2019 | | |
|  | Porphyran | | *Porphyra columbina* | | Helbert et al, 2019 | | |
|  | Pullulan | | *Aureobasidium pullulans* | | Dextra | | |
|  | Xanthan | | *Xanthomonas campestris* | | Dextra | | |
|  | Xyloglucan | | *Tamarindus indica* | | Dextra | | |
| **Secound round screening** | |  | |  | | |  |
|  | Glucosyl-(1→3)-β-D-Cellobiose | |  | | Megazyme | | |
|  | Cellobiosyl-(1→3)-β-D-Glucose | |  | | Megazyme | | |
|  | Cellotriosyl-(1→3)-β-D-Glucose | |  | | Megazyme | | |
|  | 1,4-β-D-Glucosyl-D-Mannose | |  | | Megazyme | | |
|  | 1,4-β-D-Glucosyl-D-Mannobiose | |  | | Megazyme | | |
|  | Arabinogalactan | | Larchwood | | Sigma-Aldrich | | |
|  | Cello-oligosaccharides | |  | | Prepared in house | | |
|  | Chondroïtin sulfate | |  | | TCI Europe N.V. | | |
|  | Codium CWP | | *Codium tomentosum* | | Helbert et al, 2019 | | |
|  | Ghatti gum | | *Anogeissus atifolia* | | Sigma-Aldrich | | |
|  | Karaya gum | | *Sterculia urens* | | Sigma-Aldrich | | |
|  | Poly-mannuronic acid | |  | | NTNU (Trondheim, Norway) | | |
|  | Manno-oligosaccharides | | Hydrolysed Carob galactomannan | | Prepared in house | | |
|  | Manno-oligosaccharides | | Hydrolysed Konjac glucomannan | | Prepared in house | | |
|  | Sodium alginate | | *Ascophyllum nodosum* | | CEVA (Pleubian, France) | | |
|  | Tragacanth gum | | *Astragalus gummifer* | | Dextra | | |

CM: Carboxymethyl, CWP: Cell wall polysaccharides.
