# Supplementary material for: Functional exploration of the glycoside hydrolase family GH113
Source: PLoS One. 2022 Apr 22;17(4):e0267509. doi: 10.1371/journal.pone.0267509 (PMC9032380; doi:10.1371/journal.pone.0267509)
Supplement: S2 Table — (DOCX) [file pone.0267509.s007.docx]

**S2 Table: List of synthetic substrates tested in this study**

| **Substrates** | **Provider** |
| --- | --- |
| *p*NP-α-D-galactopyranoside | Carbosynth |
| *p*NP-α-D-glucopyranoside | Carbosynth |
| *p*NP-α-D-xylopyranoside | Carbosynth |
| *p*NP-α-D-fucopyranoside | Carbosynth |
| *p*NP-α-L-fucopyranoside | Carbosynth |
| *p*NP-2-acetamido-2-deoxy-β-D-galactopyranoside | Carbosynth |
| *p*NP-2-acetamido-2-deoxy-β-D-glucopyranoside | Carbosynth |
| *p*NP-β-D-glucopyranoside | Carbosynth |
| *p*NP-β-D-glucuronide | Carbosynth |
| *p*NP-α-L-arabinofuranoside | Carbosynth |
| *p*NP-β-D-mannopyranoside | Carbosynth |
| *p*NP-β-D-xylopyranoside | Sigma-Aldrich |
| *p*NP-β-D-cellobioside | Carbosynth |
| *p*NP-β-D-maltopyranoside | Carbosynth |
| *p*NP-β-D-ribofuranoside | Carbosynth |
