# Supplementary material for: Functional exploration of the glycoside hydrolase family GH113
Source: PLoS One. 2022 Apr 22;17(4):e0267509. doi: 10.1371/journal.pone.0267509 (PMC9032380; doi:10.1371/journal.pone.0267509)
Supplement: Attachment — Submitted filename: Response to reviewers.docx [file pone.0267509.s009.docx]

Thank you for the reviewing of our revised manuscript. Here are the answers to the few points raised in the revisions:

Reviewer #1: Couturier et al have very well amended the manuscript on the function of GH113 representatives in the phylogenetic tree. A few small points to be considered are listed below.

1. Consider if there is sufficient details on the material (powder, milling, particle size – if possible) and suspension in case of insoluble polysaccharide substrates

Both insoluble polysaccharides used in our study are commercial ones (available from Megazyme), and all available documentation is easily accessible through their website. We do not have any other information that would be relevant to the article.

2. Page 4, line 79. Is the attack of different enzymes possible from the non-reducing or the reducing end or both?

Both *R. intestinalis* and *F. prauznitii* GH113 are reducing-end mannosidases and this was added in the manuscript, l. 76.

3. Page 5, line 90, correct to “bacterium”. Note also that lines 93-94 contain a repeated text from just before

Both points corrected, thank you.

4. Page 5, line 96, perhaps write “..from the GH5_40 subfamily.”

Corrected.

5. Please write throughout GenBank (not Genbank)

Corrected.

6. Page 7, line 140, perhaps write “..final protein concentrations were between approximately 2 etc..”

Corrected.

7. Page 9, line 178, replace “beta” with the Greek letter “β"

Corrected.

8. Page 15, line 326: would you be able to give the enzyme concentration range in molar units rather than weight?

Yes, the corresponding molar concentrations were added to the manuscript, l. 338.

9. I wonder if it is best to have the section “Conclusion” after Materials and Methods. Perhaps if before is possible that seems more reasonable.

Yes, done.
